# Selective constraint, background selection, and mutation accumulation variability within and between human populations

**DOI:** 10.1186/1471-2164-14-495

**Published:** 2013-07-23

**Authors:** Alan Hodgkinson, Ferran Casals, Youssef Idaghdour, Jean-Christophe Grenier, Ryan D Hernandez, Philip Awadalla

**Affiliations:** 1 Sainte Justine Research Centre, Department of Pediatrics, University of Montreal, 3175 Chemin de la Cote-Sainte-Catherine, Montreal H3T 1C5, Canada; 2 Department of Bioengineering and Therapeutic Sciences, University of California San Francisco, 1700 4th Street, San Francisco, San Francisco, CA 94158, USA

## Abstract

**Background:**

Regions of the genome that are under evolutionary constraint across multiple species have previously been used to identify functional sequences in the human genome. Furthermore, it is known that there is an inverse relationship between evolutionary constraint and the allele frequency of a mutation segregating in human populations, implying a direct relationship between interspecies divergence and fitness in humans. Here we utilise this relationship to test differences in the accumulation of putatively deleterious mutations both between populations and on the individual level.

**Results:**

Using whole genome and exome sequencing data from Phase 1 of the 1000 Genome Project for 1,092 individuals from 14 worldwide populations we show that minor allele frequency (MAF) varies as a function of constraint around both coding regions and non-coding sites genome-wide, implying that negative, rather than positive, selection primarily drives the distribution of alleles among individuals via background selection. We find a strong relationship between effective population size and the depth of depression in MAF around the most conserved genes, suggesting that populations with smaller effective size are carrying more deleterious mutations, which also translates into higher genetic load when considering the number of putatively deleterious alleles segregating within each population. Finally, given the extreme richness of the data, we are now able to classify individual genomes by the accumulation of mutations at functional sites using high coverage 1000 Genomes data. Using this approach we detect differences between ‘healthy’ individuals within populations for the distributions of putatively deleterious rare alleles they are carrying.

**Conclusions:**

These findings demonstrate the extent of background selection in the human genome and highlight the role of population history in shaping patterns of diversity between human individuals. Furthermore, we provide a framework for the utility of personal genomic data for the study of genetic fitness and diseases.

## Background

Regions of the genome that are under constraint across multiple species have previously been used to identify functional sequences in the human genome [1-3], with the idea being that areas that remain conserved over large evolutionary time scales are likely to be involved in key biological processes. Projects such as ENCODE use information about evolutionary constraint, together with laboratory techniques, to identify putative functional regions in the human genome [2,4-6] and various metrics have been developed that attempt to formalise the level of constraint at a particular site or region of the genome, sometimes including structural information, to predict the functional consequences of mutations at those sites [7-13]. Since many metrics provide a fine scaled measure of the level of constraint, they may allow identification of the most functionally important sites and guide our understanding of fundamental evolutionary processes; for example less conserved regions may be prime targets for balancing or positive selection [14,15], whereas more highly conserved sites may show signatures of negative selection [15,16].

Furthermore, within individual genomes, less highly conserved sites may point to regions that can tolerate mutations without affecting the fitness of the individual, whereas mutations at more highly conserved sites may be lethal. Indeed, numerous studies on disease phenotypes attempt to rank putative disease causing mutations by levels of constraint or predicted impact on protein structure in order to prioritise mutations for further study (for review see [17]) and it has been suggested that an individual that carries the minor allele within a population at a highly conserved site may have a greater mutation load [18,19]; an accumulation of such events across the entire genome may well impact on the overall fitness of the individual. Similarly, the frequency of an allele segregating at a site also appears to be a good indicator of functional importance, and it has been shown that variants segregating at non-synonymous sites, which are putatively functional, tend to be at lower frequency than those at synonymous sites, regardless of the function of the gene [20]. Thus, the large proportion of rare variants identified in recent studies [21,22] may have an impact on fitness.

Recent work has also sought to utilise information from an individual genome to better understand the causes of disease, and many studies have been successful in identifying causal variants on a case-by-case basis [23-26], although often only in the context of Mendelian disorders. Beyond this, ‘personal’ genomic approaches using various types of information from whole-genome sequencing, transcriptomics, proteomics and metabolomics seek to assess disease risk and tailor therapeutics [27,28]. Although in their infancy, these types of approaches are already proving successful in identifying risk factors and pre-empting symptoms through early treatment [29,30], highlighting the potential of considering individual genomes in the context of population genetics.

In this study we utilise the fine-scaled nature of one particular measure of evolutionary constraint, Genomic Evolutionary Rate Profiling (GERP), together with the substantial information now available from a large-scale genome sequencing effort, The 1000 Genomes Project, to both validate and utilize comparative and population level information to capture critical genetic events. It has previously been shown that there is a relationship between GERP and derived allele frequency (DAF) using a few individuals or considering a small proportion of the genome [18,19]. Using polymorphism data across 1,092 individuals from 14 populations in phase 1 of the 1000 Genomes Project [31] we confirm the relationship between GERP and the minor allele frequency (MAF) of a site, both in coding regions and genome-wide (see Additional file 1: Figures S1-S7). Given that we find a strong relationship between levels of evolutionary constraint and human genetic diversity, we consider how evolutionary constraint can be used to infer patterns of selection and potentially represent genetic fitness in human populations. We make three observations: 1) The depression in average MAF around coding regions is more severe for genes that are most highly conserved across species and decreases as genes become less conserved, a pattern that is repeated at conserved sites in non-coding regions. This direct evidence suggests that negative selection is the primary mechanism shaping patterns of diversity within functionally important regions of the human genome and in the surrounding sequences via background selection, 2) Effective population size correlates with patterns of allele frequencies in the regions surrounding genes, with more extreme depressions in MAF observed in populations with a smaller effective size. This suggests that selection may be less efficient in these populations, allowing more putatively deleterious alleles to segregate, which translates into higher individual mutation load 3) We detect significant differences between some individuals within populations for the number of putatively deleterious rare alleles they are carrying by comparing the distributions of constraint scores for rare alleles on an individual level. This implies that there are differences in the accumulation of putatively deleterious alleles between supposedly healthy individuals.

## Results

### Variability in constraint distinguishes modes of selection

**Figure 1:**
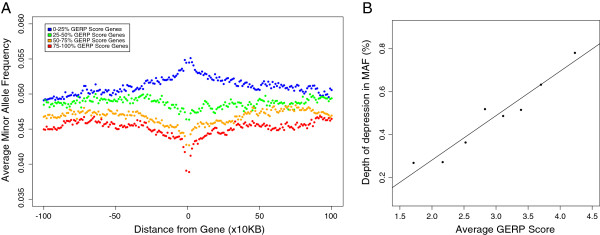
**The relationship between the average GERP score of a gene and the MAF of polymorphisms in the surrounding regions.** Genes were split into quartiles based on average GERP score and the average MAF calculated in the sequences surrounding coding regions **(A)**. The correlation between the depth of the depression in minor allele frequency and the average GERP score of genes in each of the top eight GERP score bins **(B)**.

In humans, it is known that there is a reduction in MAF around coding regions that increases further away from genes [32], however there is still some debate as to which mechanisms drive genetic diversity within a species [33]. In order to distinguish between the impact of positive and negative selection in coding regions we considered how allele frequencies in regions surrounding genes correlate with the level of sequence conservation within a gene; we observe two striking results (Figure 1A). First, the deepest depressions in MAF occur in the regions surrounding the most highly conserved genes. By splitting genes into ten groups based on average GERP score, we observe a depression in MAF around genes for the eight most conserved groups of genes (Additional file 1: Figure S8 and Table S1) and the depth of the depression in MAF correlates significantly with the average GERP score of genes in each bin (r = 0.98, p < 0.001, Figure 1B). As the reduction in diversity reflects the fraction of mutations under selection [34], and by logical extension so does the depth of the depression in MAF, these direct observations are most consistent with negative selection being the mechanism that primarily drives the distribution of alleles among individuals, with background selection affecting allele frequencies in the flanking regions of genes, since it is counter intuitive to expect more positively selected mutations at sites that have been rigorously conserved in the past. To ensure that the pattern is not driven by direct selection acting on variants falling at functional sites away from coding regions, such as regulatory elements, we repeated the analysis removing any SNPs that fall at a functionally annotated coding site or at a site with a GERP score greater than one (since sites with GERP < 1 tend to have no correlation with MAF and thus are putatively neutral, see Additional file 1: Figure S7); we observe almost identical results and the depth of depression in MAF correlates significantly with the average GERP score of genes in each bin (r = 0.97, p < 0.001, see Additional file 1: Figures S9 and S10 and Table S2). Low coverage 1000 Genomes data is known to have a higher error rate than the high coverage 1000 Genomes exome data (false discovery rate (FDR) 1.8% for low coverage, 1.6% for high coverage, see Tables S4 and S5 from [31]). However, our observation is unlikely to be a consequence of SNP calling errors for two reasons. First, it seems unlikely that a high proportion of errors would systematically cluster in certain regions. Second, in order to reduce the impact of false positive calls if any, we repeated the analysis excluding singletons, since it is at these sites where the highest proportion of error is expected (FDR = 4.4%, see Table S4 from [31]), and we observed identical results, replicating the pattern of reduced MAF around the most conserved genes (Additional file 1: Figures S11-S13).

**Figure 2:**
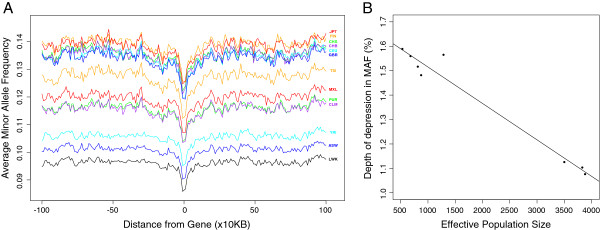
**The relationship between effective population size (*Ne*) and the MAF of polymorphisms in the regions surrounding the most conserved genes.** For genes with the highest GERP scores (top 10%), the average MAF scores surrounding genes in each population, with population codes shown in the corresponding colour to the right of each line **(A)**. The correlation between *Ne* and the depth of depression in MAF around the most highly conserved genes for old world populations that we have *Ne* data **(B)**. Population codes are as follows: Utah residents with Northern and Western European ancestry (CEU), British in England and Scotland (GBR), Toscani in Italy (TSI), Finnish from Finland (FIN), Han Chinese in Beong, China (CHB), Southern Han Chinese (CHS), Japanese in Tokyo, Japan (JPN), Yoruba in Idadan, Nigeria (YRI), Luhya in Webuye, Kenya (LWK), Americans of African Ancestry in S.W. USA (ASW), Mexican ancestry from Los Angeles, USA (MXL), Puerto Ricans from Puerto Rico (PUR) and Colombians from Medellin, Colombia (CLM).

Second, across populations the lowest overall depressions in MAF occur around the most highly conserved genes for populations with the largest effective population size (*Ne*) (Figure 2A). Resequencing in populations with larger *Ne* has discovered both more diversity in general, as well as a high number of sites with lower MAF (0.5-5%) [31,32] and in line with this we see reduced overall MAF in the regions flanking genes in these populations. However, the difference in MAF between more distal flanking regions and those adjacent to the most conserved loci is higher in populations with smaller *Ne* (Additional file 1: Figure S12, correlation between depth of depression in MAF and *Ne*: r = −0.98, p < 0.001, Figure 2B) Again, this is not driven by direct selection acting on functional sites away from coding regions; removing functionally annotated sites and those with GERP > 1 we observe almost identical results (correlation between *Ne* and depth of depression: r = −0.97, p < 0.001, see Additional file 1: Figures S15 and S16). Finally, we also observe an increase in average MAF in the sequences surrounding the least conserved genes that appears to most likely be a consequence of sequencing/mapping errors of common SNPs, since the increase in MAF is only present around multi-copy genes and is also less dramatic when SNPs out of Hardy-Weinberg equilibrium are removed (see Additional file 1: Figures S17-S19).

**Figure 3:**
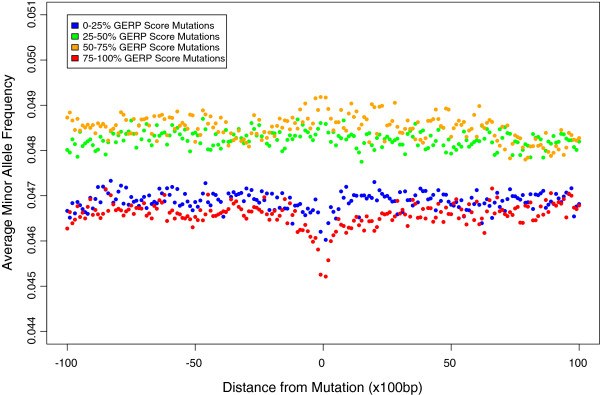
The relationship between the average GERP score of a non-coding site that is at least 200 KB away from known genes and the MAF of polymorphisms in the surrounding regions.

Non-coding regions that have been conserved over large evolutionary timescales are also likely to be functionally important, and recently noncoding transcribed elements that are not conserved across species were shown to be undergoing lineage specific purifying selection in humans [35]. To test for evidence of selection in non-coding regions at conserved sites we isolated any SNPs that are at least 200 KB from known genes. Amongst these we observe a significant decrease in average MAF in sequences surrounding SNPs at positions that are most highly conserved (p < 0.01, Mann–Whitney *U*-test), yet a depression is not present around sites with intermediate GERP scores (Figure 3). We also observe a significant decrease in average MAF in the sequences surrounding the least conserved SNP positions (p < 0.01, Mann–Whitney *U*-test). This is probably a consequence of linkage to conserved sites and is likely to be associated with the way that GERP is calculated, since we find that sites with the most negative constraint scores tend to be preferentially located adjacent to runs of conserved sites in non-coding regions (for more details see Additional file 1: Figure S20). Splitting noncoding sites into ten groups based on GERP score, we again observe a gradient in the depth of depression of average MAF in the surrounding sequences, with more conserved sites causing the deepest depression (Additional file 1: Figure S21). For the same reasons as before, these patterns are most likely driven by negative selection, and consistent with this, we also find that there is significantly less differentiation amongst all 1000 Genomes populations for non-coding SNPs with the highest GERP scores compared to those with lower GERP scores (Additional file 1: Figure S22).

### Variation in mutation accumulation within 1000 Genomes populations

It has been suggested that an individual that carries the minor allele at a conserved site may have a greater mutation load [18,19]. In line with this, we compared all individuals within a population in an attempt to identify any individuals that are carrying a higher proportion of putatively deleterious alleles. Since interspecies constraint appears to be a consistent proxy for both observed allele frequencies and inferred selection acting on alleles, we considered the potential differences in genetic fitness at the individual level by testing whether individuals have significantly different distributions of GERP scores for nonsynonymous sites at which they carry the minor allele using high-coverage exome sequencing data from the 1000 Genomes Project (average coverage 50-100×). Within each population we performed pairwise comparisons for all possible pairs of individuals and found no evidence to suggest that any individuals have a significantly different median GERP score (Mann–Whitney U tests) or significantly different distributions of GERP scores (Kolmogorov-Smirnov tests) across all comparisons (p < 0.05 after Bonferroni correction). This suggests that there are not large differences in fitness between individuals from the same population at nonsynonymous sites. However, since many alleles are shared across individuals within a population, including all observed polymorphisms in an analysis of mutation accumulation may reduce the variance in the distributions of GERP scores between individuals. Considering only rare alleles (singletons) carried by individuals within a population using high-coverage data only, we observe 22 pairs of individuals that have significantly different distributions of GERP scores (Bonferroni corrected p < 0.05, 10 pairs identified using Mann–Whitney U tests, 3 using Kolmogorov-Smirnov tests and 9 using both tests, Table 1), coming from five different populations (Colombians from Medellin, Colombia (CLM), Mexican ancestry from Los Angeles, USA (MXL), Puerto Ricans from Puerto Rico (PUR), British in England and Scotland (GBR) and Luhya in Webuye, Kenya (LWK)). Three of these populations are admixed (CLM, MXL and PUR) and therefore potentially contain individuals that are contributing alleles from different ancestral populations, however we also detect significantly different pairs of individuals that are not drawn from admixed populations.

**Table 1:** Individuals with significantly different distributions of GERP scores within populations for singletons at nonsynonymous sites

**Individual 1**	**Individual 2**	**Population**	**Mann–Whitney U p-value**	**Kolmogorov-Smirnov p-value**
HG00244	HG00253	GBR	0.03720	NS
HG01342	HG01374	CLM	0.00036	3.29e-05
HG01342	HG01112	CLM	NS	0.00657
HG01342	HG01494	CLM	NS	0.02885
HG01342	HG01274	CLM	0.02758	NS
HG01374	HG01551	CLM	0.00147	7.53e-05
HG01374	HG01550	CLM	NS	0.04320
HG01374	HG01488	CLM	0.02787	0.02099
HG01551	HG01274	CLM	0.03460	NS
NA19429	NA19321	LWK	0.02957	NS
NA19384	NA19321	LWK	0.04720	NS
NA19660	NA19741	MXL	0.04898	NS
NA19723	NA19681	MXL	0.00993	0.00853
NA19723	NA19783	MXL	0.00110	0.02363
NA19723	NA19741	MXL	0.00090	0.00834
NA19723	NA19654	MXL	0.02773	NS
HG01167	HG01072	PUR	0.00204	0.02393
HG01072	HG01108	PUR	0.00015	NS
HG01072	HG01204	PUR	0.00207	0.02961
HG01072	HG01051	PUR	0.02411	NS
HG01072	HG01052	PUR	0.00866	NS
HG01082	HG01108	PUR	0.03430	0.00019

P-values are shown for Mann–Whitney U and Kolmogorov-Smirnov tests after Bonferroni correction, unless the result is non-significant (NS).

### Variation in mutation accumulation between 1000 Genomes populations

**Figure 4:**
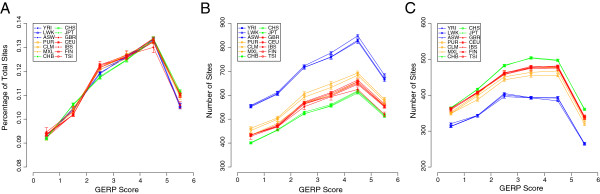
**The numbers and proportions of mutations that occur at nonsynonymous sites with different GERP scores for individuals in the 1000 Genomes populations.** For each individual, the proportion of nonsynonymous sites carrying the minor allele that fall into each GERP score bin was found and the proportions were averaged for individuals within each population in the 1000 Genomes data **(A)**. Similarly, the average distribution was found for each population using the absolute numbers of alleles at heterozygous **(B)** and homozygous derived allele (inferred from a six way primate alignment) **(C)** sites falling in each positive GERP bin. African populations are blue, admixed American populations are orange, European populations are red and Asian populations are green. Error bars denote 95% confidence intervals.

By averaging the numbers of nonsynonymous mutations that fall into each GERP category for all individuals within a population, we can make comparisons between 1000 Genomes populations for the accumulation of putatively deleterious variants. Across populations, we find very similar distributions for the proportions of minor allele sites that fall into each GERP score category (Figure 4A). However, for similar datasets that are sequenced to similar levels of coverage, the absolute number of sites may be more informative. It is known that non-African populations have lower genetic diversity compared to African populations as a consequence of the out-of-Africa bottleneck [36], and non-Africans also tend to carry a higher number of putative deleterious alleles in homozygous form due to relaxed selection [19,37]. However, little is documented about other populations. Considering heterozygous sites as a measure of genetic diversity (Figure 4B), non-African populations indeed have significantly fewer mutations falling across all constrained positions (positive GERP scores) compared to African populations (p < 0.05), but there are also significant differences between non-African populations. Individuals from admixed American populations carry more heterozygous variants at constrained sites than individuals from European populations (p < 0.05), which in turn carry more than individuals from South East Asian populations (p < 0.05). Furthermore, for derived homozygous alleles, we observe the exact opposite trend across all positive GERP score categories (Figure 4C), with individuals from South East Asia carrying the most homozygous derived alleles, followed by Europeans, Admixed Americans and then Africans (p < 0.05 in all cases). These observations are consistent with a greater relaxation of selection in South East Asian and then European populations, that is most likely a consequence of the reduced efficiency of selection in populations with smaller effective size (although varying selective effects in new environments cannot be ruled out), and increased genetic diversity caused by admixture in American populations. Finally, African and Admixed American populations tend to carry a higher number of within population singletons than Europeans and Asians (see Additional file 1: Figure S23), which is consistent with African populations having a higher genetic diversity and Admixed American populations containing individuals from different ancestral populations. Although singletons may impact upon the fitness of an individual, particularly since they are enriched at highly constrained sites, it is likely that these mutations are newer and selection has not had time to act to purge them from the population.

## Discussion

Since we confirm a relationship between GERP and MAF in phase 1 data from the 1000 Genomes Project, measures of constraint can be used to capture critical genetic events. Previously, Hernandez *et al.*[32] described a reduction in diversity around exons using 1000 Genomes pilot data [38] and concluded that it is at least partly consistent with background selection. Furthermore, Loehmueller *et al.*[39] inferred that background selection is shaping human diversity by comparing various genomic parameters in genic and non-genic regions. However, in both cases simulations were used to implicate background selection as the model that best fits the observed diversity data. In this study we present genome-wide empirical evidence of deeper depressions in MAF around the most conserved genes, which is most consistent with purifying selection being the primary mechanism driving allele frequencies in and around (via background selection) coding regions. As a consequence, it may be difficult to detect genuine evidence for selective sweeps and previous scans for such events, which rely on detecting reduced diversity around genes [40-42], may be contaminated with the effects of background selection. Our approach is robust to demographic effects and recombination, since we group genes by a measure that is calculated over long evolutionary time scales and thus is not affected by short term phenomenon, and also to variations in mutation rate, since we consider MAF rather than SNP density in the regions surrounding genes.

We also observe similar patterns in sequences surrounding the most conserved sites in non-coding regions and although Drake *et al.*[43] have previously shown that alleles segregating within conserved non-coding elements have lower MAF on average, this study provides evidence that conserved non-coding sites are not only under purifying selection but also affect alleles in the surrounding sequences via background selection to shape human diversity. The patterns are less extreme than for coding regions, but that is likely due to the spatial distribution of conserved sites being denser within a gene than in non-coding regions. Furthermore, since the variants analysed here are far away from known genes, it is unlikely that these patterns are driven by linkage to coding sites and although some regions are likely to contain functional elements such as transcription factor binding sites, these results provide yet more evidence that background selection is highly prevalent throughout the genomes of humans.

The demographic history of a population is known to impact on the frequency of alleles segregating among individuals [19,36,37]. By comparing 14 worldwide populations from the 1000 Genomes Project we can measure fine-scaled differences in the effect that demography has on the efficiency of selection. First, we observe differences in the depression in MAF around the most conserved genes for different populations that correlates with *Ne* and subsequently it is likely that deleterious alleles are segregating more readily in populations with a smaller effective size. Over time, selection has been more efficient in removing deleterious variants in populations with larger effective size, such as those from Africa, and this is clear from the reduced MAF of variants segregating in all parts of the genome in African populations (for example see Figure 2A) and the fact that African populations carry fewer putatively deleterious homozygous derived alleles (see Figure 4C). However, it is possible that there is now more potential for selection to act on variants in populations with smaller effective size, since they are at higher frequency due to past demographic events. As a consequence, perhaps as a result of recent population expansion or even a changing environment, it is possible that previously freely segregating alleles are now being driven to lower frequency in regions close to conserved genes, leading to a larger difference in the depression in MAF for populations with a smaller *Ne*. Second, by considering the average distribution of GERP scores for mutations segregating in each population we find differences across 1000 Genomes populations for the entire positive GERP score range. In general, we find that populations with smaller *Ne* tend to carry fewer heterozygous alleles, but have accumulated more variants in homozygous form. The implications for fitness depend highly on the level of penetrance at functionally important sites; if the majority of mutations are dominant, individuals from populations with more heterozygous mutations will have a higher genetic load, yet if the majority of mutations act in a recessive manner, which seems most likely since alleles at putatively damaging sites tend to persist in the population in the face of selective pressures, individuals from populations carrying more homozygous derived alleles may carry more load.

Finally, measures of constraint can also be used to classify individual genomes by the accumulation of mutations at functional sites. Within populations we infer very little variation in the accumulation of deleterious mutations across individuals when considering all nonsynonymous variants. However, it has been suggested that rare alleles drive more of the differences in phenotype between individuals, an idea that is supported by a large fraction of ‘missing heritability’ in many genome-wide association studies that focus mostly on common variation (for a discussion on this see [44]). Considering only rare alleles we detect a number of significantly different pairs of individuals that show a difference in the distributions of putatively deleterious alleles they are carrying. Although these only represent a small fraction of the total number of pairs of individuals compared across all populations, it shows that the framework of comparing distributions of constraint scores on the individual level is able to detect significant differences, even for supposedly ‘healthy’ individuals from the 1000 Genomes populations. Additionally, our approach to detect differences is conservative and may only detect the most extreme differences in mutation accumulation. First, the statistical thresholds applied in our study are likely overly conservative given that the pairwise comparisons of individual distributions of GERP scores are not independent. Second, it is also possible that by limiting this analysis to high-coverage exome data we are not accounting for the effects exerted by sites further away from the exons that may contribute further to fitness differences between individuals. Third, we predict that individual differences in the distributions of putatively deleterious variants are likely to be higher in diseased groups. As such, individuals that, for example, develop complex diseases may be more likely to come from the tails of the distribution.

## Conclusions

Using polymorphism data to infer fitness is not a new idea [45-47], however by confirming the relationship between interspecific constraint and MAF it allows us to make inferences about key genetic processes and gain a greater insight into how selection operates in human populations. By doing so we have highlighted the extent of background selection across the human genome and the interplay of selection and demographic history in shaping human diversity. Furthermore, the utility of this relationship also allows us to make predictions about disease and fitness based on a score that is not dependent on having population level data. By applying this method and using a stringent statistical threshold we were able to detect differences between ‘healthy’ individuals from the 1000 Genomes populations. Subsequently, it may be promising to consider the constraint profiles of individuals with complex diseases, a strategy which may be effective in capturing the signatures of damaging mutations that don’t necessarily occur at the same sites for a given phenotype (and are thus missed by more traditional approaches), but instead act in a cumulative manner across the genome. This approach is a useful first step to characterise the nature of mutation load on an individual level, and may have important implications in studying human fitness and disease.

## Methods

### Data

Phase 1 data from the 1000 Genomes Project were downloaded from the 1000 genomes ftp site (http://ftp-trace.ncbi.nih.gov/1000genomes/ftp/) and consists of 1092 individuals from 14 populations. 1000 genomes data used here consists of two types: whole-genome low coverage data that is sequenced to an average depth of 2-6× and high coverage exon-targeted data that is sequenced to an average coverage of 50-100×. The false discovery rate of exome and non-coding SNPs is 1.6% and 1.8% respectively [31]. Details on 1000 Genomes populations, sequencing protocol, snp calling, and validation can be found in the 1000 Genomes pilot [38] and phase 1 [31] publications. Low coverage variant call format (vcf) files were used to calculate allele frequency data across all, and within each, population. For high coverage exome data, SNPs falling within targetted exons were extracted from exome vcf files and allele frequency data was collected only for these sites. Throughout the analysis, sites were annotated using the SeattleSeq SNP Annotation tool (http://snp.gs.washington.edu/SeattleSeqAnnotation134/). GERP++ (referred to as GERP in the main text) scores, which measure evolutionary constraint based on the number of substitutions in orthologous sequences of up to 34 mammalian species [9,13], and elements were downloaded from the Sidow laboratory website (http://mendel.stanford.edu/SidowLab/downloads/gerp/index.html). Throughout our analyses when GERP scores are directly compared to MAF we exclude any sites with a GERP score of exactly 0; these represent sites where there are less than three species that could be aligned in the calculation of the GERP score [8] and are therefore uninformative. Throughout the analysis, whenever multiple distributions are compared we use a Bonferroni correction for multiple testing.

### Polymorphism patterns in coding and non-coding regions

The genomic locations of each gene were obtained from version Hs37.2 of the CCDS dataset (http://www.ncbi.nlm.nih.gov/projects/CCDS/CcdsBrowse.cgi) as these represent a collaborative and high quality annotation of protein coding regions. To calculate the average MAF of polymorphisms surrounding coding regions, genes were sorted into quartiles based on the average GERP score per gene, which represents the sum of GERP scores for all coding sites (regardless of whether they contain a SNP), divided by the total number of coding sites, and the average MAF was calculated in one hundred non-overlapping windows of 10 kb in the sequences surrounding each group of genes, using low coverage data across all populations. The process was also repeated by splitting genes into ten groups based on average GERP score. To measure the depth of depression in MAF surrounding genes we calculated the difference between the average MAF in the windows spanning from 500 KB to 1 MB and -500 KB to -1 MB relative to the position of each gene, as it is in these regions where the average MAF appears to level off, and the lowest MAF value in the central 100 KB. To consider the patterns of polymorphism around the most highly conserved genes for each population we calculated the average MAF in regions surrounding the top 10% of genes by average GERP scores as before and calculated the depth of the depression in MAF around these genes in the same manner as described above. These values were then compared to estimates of *Ne* for as many of the old world populations as were available in a study by Mele *et al.*[48] (included estimates for the YRI, LWK, CEU, GBR, TSI, CHB, JPT and ASW populations). We excluded the IBS population from this analysis due to the small number of individuals sampled.

For non-coding regions we consider only those sites that are at least 200 KB away from a known coding region, as determined by the ensembl gene data set (http://genome.ucsc.edu/), which represents probably the most exhaustive set of coding region annotations. In these regions, SNPs were sorted by GERP score into quartiles and the average MAF was calculated in one hundred non-overlapping windows of 100 bp in the sequences surrounding each group of mutations, using low coverage data across all populations. To consider whether the decrease in average MAF around conserved and non-conserved sites is significant, we tested whether the average MAF closest to the focal sites (in the surrounding 200 bp) is significantly different to the average MAF in more distal regions (in the windows spanning from 5 kb to 10 kb and from -5 kb to -10 kb, relative to the focal SNP).

### Comparison of individuals

To compare individual GERP distributions we constructed a distribution of GERP scores for each individual by including all nonsynonymous sites where the individual carried the minor allele. We assumed that fitness was additive, and thus included the GERP score twice if an individual was homozygous for the minor allele at a given site. Examples of GERP score distributions are shown in Additional file 1: Figures S24 and S25. To compare 1000 Genomes populations, we averaged the distribution of GERP scores per individual within each population for both the proportion and the absolute number of mutations falling into each GERP bin. To compare groups of populations we again averaged the distributions of all individuals within each group. We focussed on the positive GERP score range, since it is likely that only these mutations have an impact on minor allele frequency and thus fitness (see Additional file 1). For homozygous sites we use the derived allele (inferred from a six-way primate alignment obtained from the ensembl website, http://www.ensembl.org), since using the minor allele will introduce a bias due to the different sample sizes for each continental group. To compare the average number of singletons carried by individuals between populations, we took the same approach as detail above, but randomly selected the same number of individuals from each population to ensure that a singleton represented the same MAF in each population and therefore was not biased by sample size. Again, the IBS population was excluded from this analysis due to the small number of individuals sequenced.

## Competing interests

The authors declare that they have no competing interests.

## Authors’ contributions

AH, FC, YI, RH and PA designed the study, AH and JCG performed research, AH performed analysis and AH, YI and PA wrote the paper. All authors read and approved the final manuscript.

## Supplementary Material

Additional file 1 Supplementary materials. Click here for file
