# Supplementary material for: Selective constraint, background selection, and mutation accumulation variability within and between human populations
Source: BMC Genomics. 2013 Jul 23;14:495. doi: 10.1186/1471-2164-14-495 (PMC3727949; doi:10.1186/1471-2164-14-495)
Supplement: Additional file 1 — Supplementary materials. [file 1471-2164-14-495-S1.docx]

**Supplemental Materials for:**

**Title: Selective constraint, background selection, and mutation accumulation variability within and between human populations**

Authors: Alan Hodgkinson,^1^Ferran Casals,^1^Youssef Idaghdour,^1^Jean-Christophe Grenier,^1^ Ryan Hernandez^2^ and Philip Awadalla^1^*

Affiliations: 1. Sainte Justine Research Centre, Department of Pediatrics, University of Montreal, 3175 Chemin de la Cote-Sainte-Catherine, Montreal, H3T 1C5, Canada.

2. Dept. of Bioengineering and Therapeutic Sciences, University of California San Francisco, 1700 4th Street, San Francisco, CA, 94158, USA.

*Correspondence: philip.awadalla@umontreal.ca

*Validating constraint as a proxy for selection*

It has previously been shown that there is a relationship between GERP and derived allele frequency (DAF)[[1](#_ENREF_1), [2](#_ENREF_2)]. To confirm this relationship in phase 1 data from the 1000 Genomes Project, SNP sites identified in the high coverage exome dataset were sorted by MAF into 25 bins ranging from 0 to 0.5 with interval sizes of 0.02. For each bin, the average GERP score was calculated by summing GERP scores at each SNP site and diving by the total number of sites. Sites with the lowest minor allele frequencies are associated with the highest GERP scores (figure S1) and SNPs in the lowest frequency bin (MAF<0.02) have a significantly higher average GERP score than all other bins (p<0.01 in all cases). As a high proportion of SNPs in the 1000 genomes project are in the lowest frequency bin, we repeated the analysis by splitting sites with MAF below 0.02 into ten further bins; we observe similar results (figure S2). A similar relationship between MAF and GERP was observed genome-wide using all sites in the low coverage dataset (figure S3). Since the strength of selection is related to the level of reduction in genetic diversity, this implies that there is a direct relationship between the level of evolutionary constraint and fitness in human populations.

The pattern is consistent when populations are plotted separately (figure S4) or grouped by ancestry (figure S5). Interestingly, there is a significant negative correlation between the average GERP for the lowest frequency SNPs (MAF<0.02) and effective population size (*Ne*) (r=-0.96, p<0.01) for old world populations, which remains when grouping by ancestry (r=-0.99, p=0.01), highlighting both the precision of GERP in detecting differences at the population level and reinforcing the notion that selection is more efficient at removing deleterious alleles in populations with larger *Ne*. Old world ancestral groups were defined as African (YRI and LWK), European (CEU, FIN, GBR, IBS and TSI) and East Asian (JPT, CHB and CHS) and comparisons between MAF and GERP on this level were made by using a combined MAF at each site for populations in each group. To compare effective population size (*Ne*) and average GERP score for alleles with MAF<0.02, we obtained estimates of *Ne* for as many of the old world populations as were available in a study by Mele *et al.* [[3](#_ENREF_3)]; this included estimates for the YRI, LWK, CEU, GBR, TSI, CHB, JPT and ASW populations and comparisons between old world ancestral groups were made by finding the average *Ne* for populations that belonged to each group. To ensure that the relationship between the average GERP score in the lowest allele frequency bin and the average effective population size per ancestral group was not a consequence of sample size we re-sampled the data controlling for both the number of sites and the number of individuals in each group (figure S6). To control for the number of individuals in each group, 370 alleles were randomly resampled from each ancestral group at each coding site, since this is the lowest number of alleles sampled within one group (185 individuals). As before, sites were sorted by minor allele frequency into 25 bins ranging from 0 to 0.5 with interval sizes of 0.02, and then calculated the average GERP score for sites within each bin. We observe very similar results to when all individuals are sampled and the correlation between average GERP in the lowest allele frequency bin and effective population size remains significant (p<0.05). To control for differences in the number of sites sampled in each MAF bin, we randomly sampled 5000 sites with replacement from each group of populations within each MAF bin and then repeated the analysis as before to compare MAF and GERP. We observe similar result to when all sites are sampled and the correlation between average GERP in the lowest allele frequency bin and effective population size is significant (p<0.05).

Considering different sequence types, the average MAF is lowest for SNPs with the highest GERP scores not only at nonsynonymous sites, but also at both synonymous and intronic positions, suggesting that selection is also acting at these sites (figure S7). However the relationship between constraint and MAF is not consistent across the three sequence types (positive GERP score range, p<0.05,Kruskal-Wallis test), implying that GERP is not entirely predictive of processes that govern the frequencies of alleles on a population level. Finally, average MAFs are largely consistent across the negative GERP score range (figure S7), suggesting that these sites are probably neutral.

*Variability in constraint distinguishes modes of selection*

After grouping genes by average GERP scores we observe an increase in the average minor allele frequency surrounding the least conserved genes. There are a number of possible explanations for this increase. First, the least conserved genes may be undergoing partial selective sweeps, however this is unlikely, as sweeps should also decreased genetic diversity in the surrounding region, which we do not observe (figure S17). Second, the genes may be under balancing selection and alleles are being held at intermediate frequencies due to an advantage inferred from having a diverse population at these sites, and third, there are more mapping/sequencing errors for genes that are less well conserved. To differentiate between these possibilities, we split our genes into single and multi-copy genes and find that the peak is only present for multi-copy genes (figure S18). To split genes into multi and single copy we considered a gene to be multi copy if a paralogous gene was identified in the ensembl gene database via ensemblbiomart (http://www.ensembl.org/biomart/martview/), otherwise it was considered to be a single copy gene. We used the same number of multi-copy and single-copy genes by random sampling. We also removed SNPs that did not pass a Hardy-Weinberg threshold (p<0.01) and find that the sharpness of the peak in MAF around the least conserved genes is reduced (figure S19). Both of these results are more consistent with sequencing/mapping error of common SNPs.

To test whether the decrease in MAF around non-conserved sites is driven by linkage to more highly conserved sites we considered the average GERP score for each site flanking conserved GERP elements (runs of sites with positive GERP scores, 100bp in each direction) that were identified as being significantly conserved in the original production of GERP scores[[4](#_ENREF_4)]. We find a decrease in average GERP score that is lowest immediately adjacent to GERP elements and increases towards more distal regions (figure S20), implying that high and low GERP scores tend to cluster and that the patterns of MAF in regions surrounding the least conserved sites probably mirror the patterns surrounding the most conserved sites because of linkage. One possible explanation for this pattern is that GERP scores tend to be more extreme in regions that are most highly conserved as a consequence of being able to align more species when calculating the GERP score. Thus, any regions flanking conserved sites may also be easier to align and as such may have the capacity to accrue more substitutions between species that are used to calculate the GERP score.

To consider the levels of population differentiation in non-coding regions we split noncoding mutations by GERP score into ten bins and calculated the average combined F_ST_ across all 1000 Genomes populations for each group of sites using low coverage data and find that sites with the highest constraint scores have significantly lower average F_ST_ than all other bins (Kruskal-Wallis test, p<0.05 in all cases, figure S22).

**Figure S1:**The relationship between GERP and minor allele frequency (MAF) for all populations at coding sites. Sites were split by MAF into 25 bins and the average GERP score is calculated per group using high-coverage exome data. Error bars denote 95% confidence intervals.

**Figure S2:**The relationship between GERP and MAF for all populations at low frequency coding sites. As a high proportion of SNPs in the 1000 genomes project are in the lowest frequency bin, sites with MAF below 0.02 were split into ten further bins and the average GERP per bin was calculated.Error bars denote 95% confidence intervals.

**Figure S3**: The relationship between GERP and minor allele frequency (MAF) for all populations genome-wide. Sites were split by MAF into 25 bins and the average GERP score is calculated per group using low-coverage data.Error bars denote 95% confidence intervals.

**Figure S4**: The relationship between GERP and MAF at coding sites for each population. The relationship is consistent with patterns observed when grouping populations together.

**Figure S5**: The relationship between GERP and MAF at coding sites for groups of populations.Populations were grouped by ancestry, MAF calculated within each group and then sites were split into 25 bins based on MAF, with the average GERP calculated per bin.Error bars denote 95% confidence intervals.

A B

**Figure S6**: The relationship between MAF and GERP in coding regions for ancestral groups after controlling for different numbers of individuals (a) and SNPs in each group (b).

**Figure S7**: The relationship between MAF and GERP at coding sites for different sequence types. Sites were grouped by sequence type, split into bins based on GERP score (bins of size one) and the average MAF calculated per bin - MAF varies significantly between nonsynonymous and both intronic and synonymous sites for each positive GERP score bin greater than one (p<0.01 in all cases). Error bars denote 95% confidence intervals.

**Figure S8**: Average MAF in regions surrounding genes. Genes are split into ten groups based on average GERP score per gene and the average MAF of SNPs in non-overlapping windows of 10KB is shown. There is a depression in average MAF surrounding the eight most conserved groups, details on which are shown in table S1.

**Figure S9**: Average MAF in regions surrounding genes after removing sites annotated as coding and sites with GERP>1. Genes are split into ten groups based on average GERP score per gene and the average MAF of SNPs in non-overlapping windows of 10KB is shown. There is a depression in average MAF surrounding the eight most conserved groups, details on which are shown in table S2.

**Figure S10.** The correlation between the depth of the depression in minor allele frequency and the average GERP score of genes in each of the top eight GERP score bins after coding sites and those with GERP>1 have been removed (r=0.97, p<0.001).

**Figure S11.** The relationship between the average GERP score of a gene and the MAF of polymorphisms in the surrounding regions after singletons have been removed. Genes were split into quartiles based on average GERP score and the average MAF calculated in the sequences surrounding coding regions.

**Figure S12.** The relationship between the average GERP score of a gene and the MAF of polymorphisms in the surrounding regions after singletons have been removed. Genes were split into ten groups based on average GERP score and the average MAF calculated in the sequences surrounding coding regions.

**Figure S13.** The correlation between the depth of the depression in minor allele frequency and the average GERP score of genes in each of the top eight GERP score bins after singletons have been removed (r=0.96, p<0.001).

**Figure S14**: The depression in MAF around the most conserved genes (top 10% of genes sorted by average GERP score) for each population. 95% confidence intervals are shown.

**Figure S15**: The average MAF of SNPs in the regions surrounding the most conserved genes, split into non-overlapping windows of 10KB. Coding sites and those with GERP>1 have been removed and each line shows a different population (population codes indicated on the right).

**Figure S16.** The correlation between *Ne* and the depth of depression in MAF around the most highly conserved genes for old world populations that we have *Ne* data. Coding sites and those with GERP>1 have been removed.

**Figure S17**: SNP density in regions surrounding genes. Genes are split into quartiles based on average GERP score per gene and the number of SNPs in non-overlapping windows of 10KB is shown. Since there is increased SNP density around the least conserved genes, it is unlikely that they are undergoing partial selective sweeps as this process should reduce genetic diversity in the surrounding regions.

A

B

**Figure S18**: Average MAF in regions surrounding (a) multi-copy and (b) single-copy genes.

**Figure S19**: Average MAF in regions surrounding genes for SNPs that pass a Hardy-Weinberg filter (p>0.01).

**Figure S20**: Average GERP per site for regions flanking conserved GERP elements.

**Figure S21**: Average MAF in the sequences surrounding non-coding sites.In regions at least 200kb away from a known coding sites, SNPs were sorted by GERP score into ten bins and the average MAF was calculated in one hundred non-overlapping windows of 100bp in the sequences surrounding each group of mutations, using low coverage data across all populations.

**Figure S22**: Average F_ST_ for SNPs in noncoding regions. SNPs are grouped into ten bins based on GERP score and the average F_ST_ is calculated for each group. Although grouping noncoding sites together potentially masks interesting signals at single sites and introduces a lot of noise, the signal is sufficiently strong for us to detect significant differences at the most highly conserved sites that are consistent with negative selection. Error bars represent 95% confidence intervals calculated by bootstrapping.

**Figure S23.** The numbers of within population singletons that occur at nonsynonymous sites with different GERP scores for individuals in the 1000 Genomes populations. The average distribution was found for each population using the absolute numbers of singletons falling in each positive GERP bin. African populations are blue, admixed American populations are orange, European populations are red and Asian populations are green. Error bars denote 95% confidence intervals.

**Figure S24**: An example of the distributions of GERP scores per individual at nonsynonymous sites. Each line represents a single individual from the GBR population. Other populations are similar to the distributions shown here.

a) b)

**Figure S25**: Distributions of GERP scores per individual for singletons at nonsynonymous sites. Each line represents an individual from (a) the CLM population and (b) the MXL population – two of the three populations that have significant outliers in both Kruskal-Wallis and Kolmogorov-Smirnov tests.

**Table S1:** The average GERP score for genes in each bin and the corresponding depression in minor allele frequency in the surrounding regions. In the vast majority of comparisons, genes with higher constraint scores are associated with significantly larger reductions in MAF in the surrounding sequences.

| Gene bin | Average GERP score per gene | Depression in MAF | 95% Confidence interval |
| --- | --- | --- | --- |
| 0-10% | -0.15 | -0.686% | (-0.708%, -0.665%) |
| 10-20% | 1.04 | -0.444% | (-0.459%, -0.429%) |
| 20-30% | 1.71 | 0.125% | (0.107%, 0.143%) |
| 30-40% | 2.17 | 0.254% | (0.237%, 0.271%) |
| 40-50% | 2.52 | 0.364% | (0.351%, 0.376%) |
| 50-60% | 2.83 | 0.519% | (0.503%, 0.535%) |
| 60-70% | 3.11 | 0.487% | (0.469%, 0.505%) |
| 70-80% | 3.39 | 0.515% | (0.500%, 0.530%) |
| 80-90% | 3.70 | 0.632% | (0.620%, 0.645%) |
| 90-100% | 4.23 | 0.781% | (0.766%, 0.795%) |

**Table S2:** The average GERP score for genes in each bin and the corresponding depression in minor allele frequency in the surrounding regions, with coding sites and those with a GERP>1 removed. In the vast majority of comparisons, genes with higher constraint scores are associated with significantly larger reductions in MAF in the surrounding sequences.

| Gene bin | Average GERP score per gene | Depression in MAF | 95% Confidence interval |
| --- | --- | --- | --- |
| 0-10% | -0.15 | -0.656% | (-0.682%, -0.635%) |
| 10-20% | 1.04 | -0.413% | (-0.430%, -0.397%) |
| 20-30% | 1.71 | 0.153% | (0.134%, 0.173%) |
| 30-40% | 2.17 | 0.265% | (0.246%, 0.283%) |
| 40-50% | 2.52 | 0.372% | (0.359%, 0.386%) |
| 50-60% | 2.83 | 0.536% | (0.518%, 0.554%) |
| 60-70% | 3.11 | 0.492% | (0.471%, 0.512%) |
| 70-80% | 3.39 | 0.501% | (0.485%, 0.518%) |
| 80-90% | 3.70 | 0.613% | (0.599%, 0.626%) |
| 90-100% | 4.23 | 0.752% | (0.735%, 0.768%) |

**References**

1. Cooper GM, Goode DL, Ng SB, Sidow A, Bamshad MJ, Shendure J, Nickerson DA: **Single-nucleotide evolutionary constraint scores highlight disease-causing mutations**. *Nature methods* 2010, **7**(4):250-251.

2. Goode DL, Cooper GM, Schmutz J, Dickson M, Gonzales E, Tsai M, Karra K, Davydov E, Batzoglou S, Myers RM *et al*: **Evolutionary constraint facilitates interpretation of genetic variation in resequenced human genomes**. *Genome research* 2010, **20**(3):301-310.

3. Mele M, Javed A, Pybus M, Zalloua P, Haber M, Comas D, Netea MG, Balanovsky O, Balanovska E, Jin L *et al*: **Recombination gives a new insight in the effective population size and the history of the old world human populations**. *Molecular biology and evolution* 2012, **29**(1):25-30.

4. Davydov EV, Goode DL, Sirota M, Cooper GM, Sidow A, Batzoglou S: **Identifying a high fraction of the human genome to be under selective constraint using GERP++**. *PLoS computational biology* 2010, **6**(12):e1001025.
